# Severe fever with thrombocytopenia syndrome can masquerade as hemorrhagic fever with renal syndrome

**DOI:** 10.1371/journal.pntd.0007308

**Published:** 2019-03-29

**Authors:** Rui Qi, Xiang-rong Qin, Ling Wang, Hui-ju Han, Feng Cui, Hao Yu, Jian-wei Liu, Xue-jie Yu

**Affiliations:** 1 Wuhan University School of Health Sciences, Wuhan, China; 2 State Key Laboratory of Virology, Wuhan University, Wuhan, China; 3 Zibo Center for Disease Control and Prevention, Zibo City, China; 4 Fudan University School of Medicine, Shanghai, China; Faculty of Science, Ain Shams University (ASU), EGYPT

## Abstract

**Background:**

Severe fever with thrombocytopenia syndrome (SFTS) is an emerging viral hemorrhagic fever with a high fatality rate and high frequency of person-to-person transmission and is caused by SFTSV, a tick-borne *Phlebovirus*. Because SFTS has similar clinical manifestations and epidemic characters (such as spatial and temporal distributions) with hemorrhagic fever with renal syndrome (HFRS) in China, we reason that SFTS patients might be misdiagnosed as HFRS.

**Methodology/principal findings:**

Acute-phase sera of 128 clinically diagnosed HFRS patients were retrospectively analyzed for *Hantavirus* IgM antibodies with ELISA. *Hantavirus*-negative patients’ sera were further analyzed for SFTSV IgM antibodies with ELISA. ELISA showed that 73 of 128 (57.0%) of clinically diagnosed HFRS patients were IgM antibody positive to Hantaviruses. Among the 55 *Hantavirus*-IgM negative patients, four (7.3%) were IgM antibody positive to SFTSV. The results indicated that the four SFTS patients were misdiagnosed as HFRS. The misdiagnosed SFTS patients had clinical manifestations common to HFRS and were unable to be differentiated from HFRS clinically.

**Conclusions:**

Our study showed that SFTS patients could be clinically misdiagnosed as HFRS. The misdiagnosis of SFTS as HFRS causes particular concern because it may increase the risk of death of SFTS patients and person-to-person transmission of SFTSV without proper care for and isolation of SFTS patients.

## Author summary

SFTS were clinically misdiagnosed as HFRS. It could cause particular concern in China. Physicians could not rely heavily on the exposure history. Both SFTS and HFRS patients are treated based on the clinical diagnosis in China. Laboratory confirmation of both diseases is not performed in clinical hospitals and the patients’ blood was usually submitted to a local or provincial center for disease control and prevention. In most cases the confirmation diagnosis is to provide retrospective information rather than to guide clinical therapy. Therefore, physicians need to carefully differentiate SFTS and HFRS patients because the fatality of SFTS is much higher than HFRS and SFTS is easily spread from person to person by contacting infected blood or even through aerosol.

## Introduction

Severe fever with thrombocytopenia syndrome (SFTS) is an emerging hemorrhagic fever and its pathogen SFTS virus (SFTSV) was first discovered in China in 2010 [1]. SFTSV is transmitted by tick bite and frequently spread from person-to-person [2–4]SFTSV is a negative-strand RNA virus belonging to the *Phlebovirus* genus of the family *Bunyaviridae* [1]. SFTS is a viral hemorrhagic fever with higher frequency of hemorrhagic signs than Ebola virus disease or dengue [5, 6]. SFTS caused attention and fear because of high case fatality and its potential to cause family cluster infection in China. The case fatality rate of SFTS is very high, ranging from 12 to 30% in China and 22 to 30% in South Korea and Japan [1, 5, 6]. SFTS was first recognized as a family cluster of infection in central China and family clusters SFTS has been reported in China and South Korea even after discovery of SFTSV [2, 3, 7–16]. Since discovery of SFTSV in 2010, more than 2000 confirmed SFTS patients were reported annually in China [17].

Hemorrhagic fever with renal syndrome (HFRS) is caused by *Hantavirus*, which are RNA viruses belonging to the genus *Hantavirus* of the family *Bunyaviridae*. Hantaviruses are carried and transmitted by rodents. Humans acquire infection after direct contact with infected rodents or their excreta, which most likely occurs by inhaling virus-contaminated aerosols [18]. HFRS occurs worldwide with about 90% of HFRS cases having been reported in China [19]. In the past decades, more than 10,000 of HFRS cases were reported annually, and the fatality rate was about 1% in China [20]. HFRS has a high incidence rate (average 1.96 cases/100,000 persons) in Zibo City, Shandong Province [21, 22]. We have also demonstrated SFTSV infection is present in patients and in domesticated and wild animals in Zibo City, Shandong Province [23, 24]. These studies indicated Zibo City was hard hit by *Hantavirus* and SFTSV. Because SFTS and HFRS have similar clinical manifestations and epidemic features, we reasoned that SFTS might be clinically misdiagnosed as HFRS. The aim of this study is to determine whether SFTS was clinically misdiagnosed as HFRS by testing IgM antibodies against Hantavirus and SFTSV in acute sera of those clinically diagnosed HFRS patients.

## Methods

### Ethics statement

The study was reviewed and approved by the ethics committees of Wuhan University and Zibo Center for Disease Control and Prevention.

### Data source

Acute serum samples of clinically diagnosed HFRS patients from 2013 to 2014 were retrospectively obtained from a local center for disease control and prevention in Zibo City, Shandong Province, China. Zibo City is a prefecture-level city which consists of 6 districts and 3 counties distributed over 5,938 km^2^ of land. The total population during the 2010 census was 4.53 million, of whom 900,000 persons were farmers. The city is about 42% mid-sized mountains, 30% hills, and 28% plains. Acute serum samples of clinically diagnosed HFRS patients were collected through physicians in hospitals which were under the jurisdiction of Zibo City. When a patient was diagnosed as HFRS according to the case definition, serum sample would be collected at once. Epidemiological and clinical data was also obtained including demographic information (age, sex, and occupation), clinical characteristics (time of disease onset and clinical features) and epidemiological exposure to rodents and ticks.

### Clinical case definition of HFRS

Clinical cases included in this study were diagnosed according to the criteria of the Chinese Center for Disease Control and Prevention for HFRS surveillance program (http://www.chinacdc.cn/jkzt/crb/lxxcxr/cxrjc/200508/t20050810_24189.htm). In the diagnosis criteria, a suspected HFRS patient meets the following conditions: initial symptoms such as a sudden onset of fever (>38 °C) and body ache plus orbital problems (such as orbital pain) or skin flushing (redness of the face, neck or upper chest) or bleeding disorders (such as conjunctival injection, petechial, axillary hemorrhage). A suspected case with symptoms plus abnormal laboratory tests (such as thrombocytopenia and proteinuria) or clinical phases (such as hypotensive, oliguric and polyuric phases) is defined as a clinically diagnosed case. A confirmed HFRS case is a clinically diagnosed patient plus one of the following conditions: serum IgM antibody positive to Hantavirus, IgG antibody seroconversion or 4-fold increases, or RT-PCR positive to Hantavirus RNA. IgM antibody test was used in this study to have a confirmed diagnosis.

### Detection of IgM antibodies to Hantaviruses and SFTSV

The acute-phase of patients’ sera was tested for anti-hantavirus IgM and anti-SFTSV IgM antibodies with ELISA. Anti-hantavirus IgM was detected by using a HFRS-IgM ELISA Kit (Wantai Biological Pharmacy, Beijing, China) which coated with anti-Human IgM (μ-chain specific) antibodies to capture IgM antibodies in the serum and detected with HRP-conjugated Hantavirus antigen.

The hantavirus-negative patients’ sera were further analyzed for anti-SFTSV IgM antibodies by using a SFTSV-IgM ELISA Kit (Xinlianxin Biotech CO., LTD, Wuxi, Jiangsu, China) according to the manufacturer’s instructions. Briefly, 96-well plates were coated with anti-Human IgM antibodies and undiluted human serum was used for detection of serum IgM, with HRP-conjugated recombinant SFTSV-proteins antigens used to visualize the results. The optical density (OD) value of each ELISA was read using an ELISA reader at 450 nm.

The proportions were used for descriptive statistics of characteristics and clinical features of patients and the rates for the detection of HFRS and SFTS. Statistical analysis was performed with R software, and pie charts and histograms were plotted by the R Graphics package.

## Results

### Laboratory diagnosis of the patients

The acute sera of 128 clinically diagnosed HFRS patients were first tested by ELISA for Hantavirus IgM antibodies. ELISA results showed that 57.0% (73/128) of patients were seropositive to hantavirus; that among the 55 sera which were IgM negative to hantavirus IgM antibodies, 4 (7.3%) were positive to SFTSV IgM antibodies. The average days of HFRS between diseases onset and sampling was 10.2 days, ranged from 4 to 20 days; Four SFTS cases were 7, 7, 10 and 12 days, respectively.

### Demographic characteristics and exposure history

**Fig 1:**
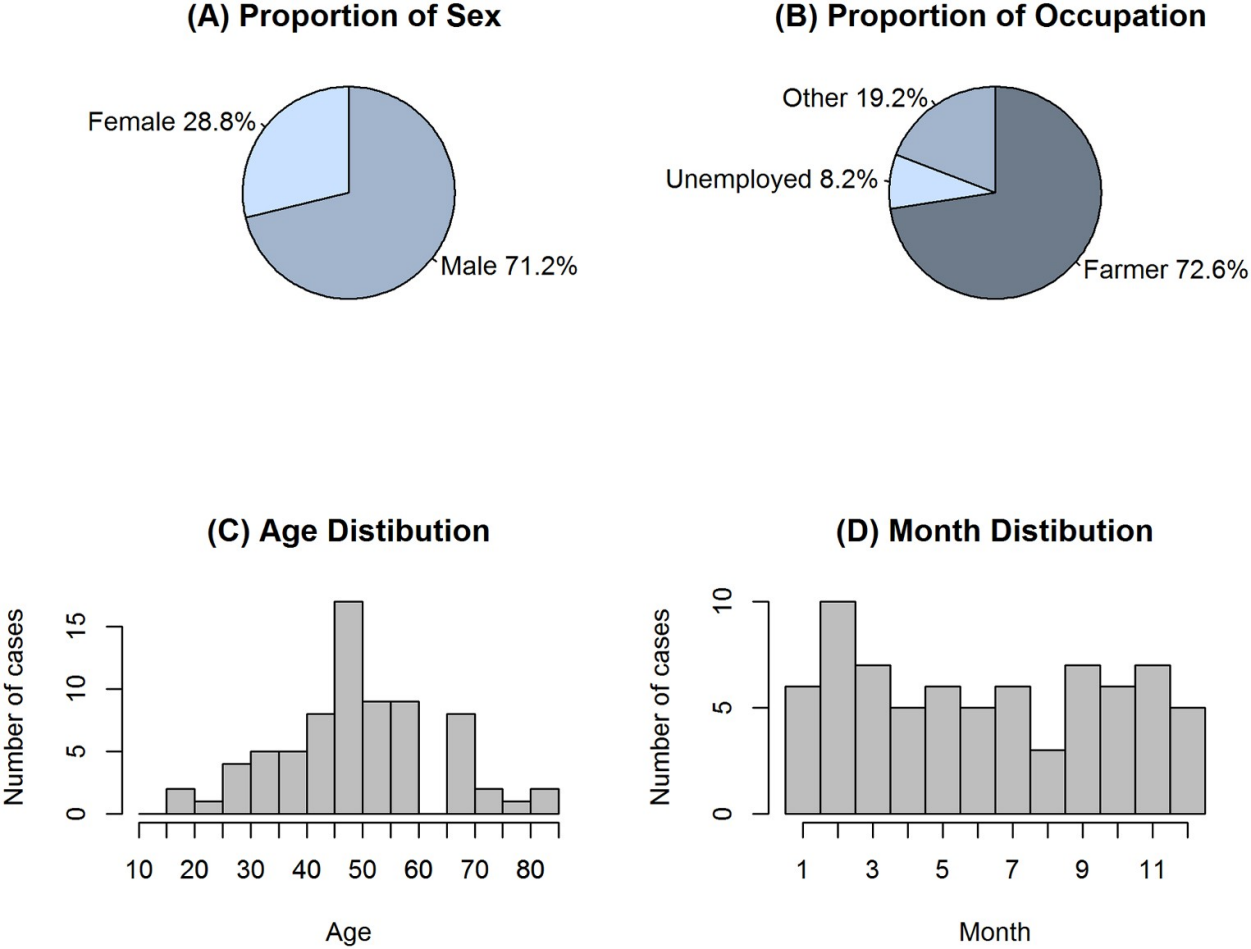
Demographic characteristics of HFRS patients. Characters of 73 laboratory-confirmed HFRS cases. (A) and (B) are pie charts of proportions of sex and occupation, respectively. (C) and (D) are histograms used to show distributions of age and time (month) of disease onset.

Among the confirmed HFRS patients 71.2% (52/73) were men, 72.6% (53/73) were farmers (The percentages of men and farmers in general population in Zibo City were 50.1% and 19.7%, respectively); 60.3% (44/74) were between 40 and 60 years of age. Temporal distribution by month of disease onset was calculated and no seasonal pattern was observed for the confirmed HFRS patients (Fig 1) and the four SFTS patients occurred in May and June three of them were male and one was female. They all were farmers aged between 47 and 69 years. At the time of enrollment, all patients were interviewed about their previous exposure history to rodents. Among the confirmed HFRS patients, 24.7% (18/73) reported a history of contact with rodents or their excreta in the last two months before disease onset. Compared with the low percentage of direct contact, a total of 65.8% (48/73) patients had observed rodents or rodent’s excreta within the patients’ home or workplace. The data indicated that HFRS patients lived in an environment with high exposure potential to rodents and their excreta. Among all clinical diagnosed 128 HFRS patients, only 3 patients reported a history of tick bites and none of the four confirmed SFTS patients had histories of tick bites.

### Clinical features of confirmed HFRS and SFTS patients

Considering SFTS patients found in clinically diagnosed HFRS cases, we compared the characteristics of ELISA-confirmed HFRS and SFTS cases (Table 1). The clinical manifestation and laboratory test results of the 4 misdiagnosed SFTS patients were hard to differentiate from the confirmed HFRS patients (Table 1). Both diseases had fever, fatigue, and headache and the most common laboratory test result of the two diseases were thrombocytopenia and proteinuria. Skin flushing (including facial flushing, reddening of neck and upper chest), oliguria, arthralgia, periorbital pain and edema, and hypotension appeared in a relatively low rate in HFRS (15–49%), but not in SFTS. Less than 10% of HFRS patients had icterus, and constipation, which were also not observed in SFTS patients (Table 1).

**Table 1:** Comparison of clinical symptoms and laboratory test of HFRS and SFTS.

	HFRS (%)	SFTS (%)
	n = 73	n = 4
Symptoms and signs		
Fever	100	100
Fatigue	89	100
Myalgia	60	100
Headache	71	75
Back pain	63	50
Arthragia	37	0
Nausea	62	100
Vomiting	41	100
Abdominal pain	41	25
Diarrhea	25	0
Constipation	1	0
Conjunctival congestion	36	50
Orbital pain	33	0
Orbital Edema^a^	18	0
Facial flushing	49	0
Redness of neck	36	0
Redness of chest	30	0
Petechiae^b^	11	0
Hematuria	33	25
Oliguria	42	0
Hypotension	15	0
Icterus	4	0
Laboratory test		
Proteinuria	100	100
Thrombocytopenia	82	100
Leukocytosis	47	25
Leukopenia	29	25

^an^ Orbital edema: conjunctival edema or periorbital edema or palpebral edema

^b^ Petechiae: petechiae or eccymosesis appeared on the palate, pharynx, or axillary skin.

Proteinuria was defined as 24-hour urinary protein > 150mg. Of the blood, thrombocytopenia was defined as platelet count < 50 x10^3^/μl; Leukocytosis was white cell count > 11x10^3^/μl and leucopenia was < 4x10^3^/μl.

## Discussion

In this study we showed that SFTS was clinically misdiagnosed as HFRS in Shandong Province, China. Both SFTS and HFRS had a broad set of similar clinical manifestations. Both diseases have a sudden onset of fever, headaches, myalgia, hemorrhagic manifestations, gastro-intestinal problems, thrombocytopenia, proteinuria, and hematuria [1, 25], which make clinical differentiation between the two diseases difficult. Previous studies indicated that SFTS patients did not have back pain and leukocytosis [26, 27]. However, our study showed that SFTS patients could have back pain and leukocytosis.

Knowledge of salient features associated with HFRS may help health care providers in diagnosis. The sample size of SFTS was 4 and we did not expect the 4 cases would be statistically significant to compare the differences between HFRS and SFTS. To better differentiate SFTS from HFRS, we further estimated pooled proportions of the clinical manifestations and laboratory findings of SFTS and HFRS from previous studies by Meta-analyses as supporting information [1, 5, 23, 24] (S1 Table). The Meta-analyses included 3200 cases of HFRS and 5046 cases of SFTS respectively which could make up for the small size of comparison. We found that SFTS are hardly to be differentiated from HFRS because they had common clinical manifestations and laboratory tests; they had no clear exposure history; and they had overlapped spatial and temporal distribution in China. SFTS mainly occurred in the summer season, while HFRS occurred year-round with two peaks in the spring and fall [17, 21, 24]. Only the following symptoms and sings may be used to differentiate SFTS and HFRS clinically, but unfortunately these symptoms and signs may not always present in SFTS or HFRS patients, respectively. Skin flushing (facial flushing, neck flushing, and chest flushing), oliguria, periorbital pain and edema, and hypotension may present in HFRS patients, but not in SFTS patients, while swollen lymph-nodes and expectoration may present in SFTS patients, but not in HFRS patients (S1 Table) [1, 27].

*Haemaphysalis longicornis* ticks have been demonstrated could serve as a vector and reservoir of SFTSV [4]. SFTSV spill over to human by tick bite. Hantaviruses which can cause HFRS are carried and transmitted by rodents, for example *Apodemus agrarius* and *Rattus norvegicus* in China. Humans acquire infection after direct contact with infected rodents or their excreta, which most likely occurs by inhaling virus-contaminated aerosols [18]. Patients for both SFTS and HFRS are farmers that reside in hilly areas infested with rodents and ticks. Our study showed that only a quarter of HFRS cases reported they had direct contact with rodents or their excreta. A previous study showed also only a quarter of SFTS cases reported a history of tick-bites [28]. Majority of HFRS and SFTS cases had no clear exposure history reported, and as a result, physicians could not rely heavily on the exposure history to differentiate the two diseases. Although most cases had no clear report of direct contact, 65.8% of patients had observed rodents or their excreta within their home or workplace, and 72.6% of cases were farmers, which indicated these patients could have significant chances to be exposed to rodents in their living environment. It has been shown that *Hantavirus* could be transmissible by inhalation of respirable droplets of saliva or urine. It would be hard to report the history of exposure when infection was caused by inhalation of pathogens. These factors would make exposure information insufficient for clinical differential diagnosis.

In this study we demonstrated that SFTS is difficult to differentiate from HFRS. Both SFTS and HFRS patients are treated based on the clinical diagnosis. Laboratory confirmation of both diseases was not performed in clinical hospitals and the patients’ blood was usually submitted to a local or provincial center for disease control and prevention. In most cases the confirmation diagnosis is to provide retrospective information rather than to guide clinical management. Therefore, physicians need to carefully differentiate SFTS and HFRS patients because the fatality of SFTS is much higher than HFRS and because SFTS is easily spread from person to person through contact with infected blood or even through aerosol [2, 3, 29].

IgM tests have proven useful and are commonly performed in diagnosis of acute infection [30]. Detection of IgM antibodies is a standard method for diagnosis of HFRS and SFTS in China as recommend by the Ministry of Health of China (http://www.chinacdc.cn/jkzt/crb/lxxcxr/cxrjc/200508/t20050810_24189.htm.). The sensitivity of HFRS-IgM kit was 98.19% and specificity was 99.28% according to the kit brochure. The specificity and sensitivity of SFTSV-IgM ELISA Kit were similar to those of the microneutralization assay and anti-SFTSV IgM exhibited no cross-reactivity with these antibodies to other closely related viruses such as Hantavirus [8]. In this cited article, SFTSV-IgM positive serum samples were diluted in 2-fold increments for detection of IgM antibodies with the same kit. Antibodies with titer as low as 1:32 still could be detected by this kit which indicated it performed well. The aim of the study was to determine whether SFTS was clinically misdiagnosed as HFRS. So, the specificity of SFTSV-IgM ELISA Kit was important to avoid false-positive results. We have tested 90 negative sera stored in our lab collected from healthy persons to evaluate the specificity of SFTSV-IgM ELISA Kit. None of them was positive which indicated the specificity could be as high as to 100%. We used ELISA to detect IgM antibodies to confirm hantavirus and SFTSV infection rather than viral RNA detection in this study because the sera have been frozen and thawed several times, which may diminish or destroy the viral RNA and we do not have sufficient quantities of samples to do both ELISA and nucleic acid detection. Our study was a retrospective analysis. It has been a few years since these samples were collected from patients between 2013 and 2014. There was not any sequential sampling of the patients, and not obtained any other sequentially information during the hospitalization. In addition, serological diagnosis might include IgG-conversion detection. However, we do not have convalescent sera to confirm whether the remaining Hantavirus- and SFTSV-IgM negative patients were seroconverted to hantaviruses or SFTSV. Though some HFRS patients might not be confirmed, it was a reality that SFTS had been found in patients managed and treated as HFRS.

In conclusion, our study showed that SFTS patients could be clinically misdiagnosed as HFRS. Both SFTS and HFRS have similar clinical manifestations, but the following manifestations including skin flushing (facial flushing, neck flushing, and chest flushing), oliguria, periorbital pain, periorbital edema, hypotesion, and/or icterus only presented in HFRS and swollen lymph nodes and expectoration are mainly present in SFTS. The misdiagnosis of SFTS as HFRS causes particular concern because it may increase the risk of death of SFTS patients and person to person transmission of SFTSV without proper care for SFTS patients. To avoid misdiagnosis, rapidly and reliable diagnostic approaches like ELISA should be widely applied in the clinical settings.

## Supporting information

S1 Table Meta-analysis of clinical symptoms and laboratory test results of HFRS and SFTS. Legend: The clinical symptoms, clinical signs and laboratory test results of HFRS and SFTS were analyzed by meta-analysis of previous studies with the following method: Firstly, we identified publications studying the clinical characteristics of Chinese HFRS and SFTS, respectively. Our principal summary data were the total number of confirmed cases together with the number or rate of clinical characteristics (symptoms and laboratory test results). Finally, 12 studies (3200 cases) for HFRS and 37 studies (5046 cases) for SFTS were included. Random-effect models were fitted to generate estimate of pooled proportion of each clinical characteristic. To have better statistical properties, the raw proportions were first logit-transformed. All analysis was run with R software and the metafor package.
(DOCX)
