# Supplementary material for: Severe fever with thrombocytopenia syndrome can masquerade as hemorrhagic fever with renal syndrome
Source: PLoS Negl Trop Dis. 2019 Mar 29;13(3):e0007308. doi: 10.1371/journal.pntd.0007308 (PMC6457554; doi:10.1371/journal.pntd.0007308)
Supplement: S1 Table — Legend: The clinical symptoms, clinical signs and laboratory test results of HFRS and SFTS were analyzed by meta-analysis of previous studies with the following method: Firstly, we identified publications studying the clinical characteristics of Chinese HFRS and SFTS, respectively. Our principal summary data were the total number of confirmed cases together with the number or rate of clinical characteristics (symptoms and laboratory test results). Finally, 12 studies (3200 cases) for HFRS and 37 studies (5046 cases) for SFTS were included. Random-effect models were fitted to generate estimate of pooled proportion of each clinical characteristic. To have better statistical properties, the raw proportions were first logit-transformed. All analysis was run with R software and the metafor package. (DOCX) [file pntd.0007308.s001.docx]

**S1 Table.** **Meta-analysis of clinical symptoms and laboratory test results of HFRS and SFTS**

|  | Pooled proportion % (95%CI) | |
| --- | --- | --- |
|  | HFRS | SFTS |
| Symptoms and signs |  |  |
| Fever | 99 (98-100) | 98 (97-99) |
| Fatigue | 98 (84-100) | 84 (78-88) |
| Dizziness | 56 (47-65) | 25 (21-30) |
| Chill | 69 (41-88) | 47 (31-65) |
| Myalgia | 67 (44-84) | 58 (48-67) |
| Headache | 76 (66-84) | 38 (30-46) |
| **Back pain^a^** | 66 (56-75) | NA |
| Arthragia | 32 (21-45) | 20 (8-43) |
| Anorexia | 96 (90-99) | 80 (72-86) |
| Nausea | 76 (67-83) | 56 (49-63) |
| Vomiting | 60 (51-68) | 41 (35-47) |
| Abdominal pain | 44 (29-61) | 27 (18-38) |
| Diarrhea | 27 (21-33) | 37 (31-43) |
| **Constipation** | 7 (1-26) | Rarely reported |
| Conjunctival congestion | 55 (43-66) | 11 (5-23) |
| **Orbital pain** | 36 (27-46) | NA |
| **Orbital edema**^b^ | 33 (21-48) | NA |
| **Facial flushing** | 51 (39-63) | NA |
| **Redness of neck** | 38 (20-61) | NA |
| **Redness of chest** | 33 (17-54) | NA |
| **Hypotension** | 28 (17-42) | NA |
| **Oliguria** | 48 (39-58) | Rarely reported |
| **Icterus** | 6 (4-9) | Rarely reported |
| Petechiae^c^ | 56 (43-69) | 25 (21-30) |
| Hematuria | 28 (18-41) | 33 (19-50) |
| Melena | 6 (1-30) | 11 (8-17) |
| Hemoptysis | NA^d^ | 5 (3-8) |
| Cough | 16 (12-21) | 37 (30-45) |
| **Expectoration** | NA | 28 (19-41) |
| **Lymphadenopathy** | Rarely reported^e^ | 42 (36-48) |
| Lab test | | |
| Proteinuria | 96 (91-100) | 69 (60-76) |
| Thrombocytopenia | 79 (70-86) | 94 (90-97) |
| **Leukocytosis** | 71 (54-83) | NA |
| Leukopenia | 7 (2-21) | 85 (79-89) |

**^a^** Bold characters indicated symptoms and signs that were specific to either HFRS or SFTS.

^b^ Orbital edema: conjunctival edema or periorbital edema or palpebral edema.

^c^ Petechiae: petechiae or eccymosesis appeared on the palate, pharynx, or axillary skin.

^d^ NA: Not be reported in any included studies.

^e^ Rarely reported: reported in less than 3 studies and proportion was smaller than 5%.
